# Marriage and divorce after military deployment to Afghanistan: A matched cohort study from Sweden

**DOI:** 10.1371/journal.pone.0207981

**Published:** 2019-02-01

**Authors:** Carl-Martin Pethrus, Johan Reutfors, Kari Johansson, Kristian Neovius, Jonas Söderling, Martin Neovius, Gustaf Bruze

**Affiliations:** 1 Clinical Epidemiology Unit, Department of Medicine Solna, Karolinska Institutet, Karolinska University Hospital, Stockholm, Sweden; 2 Cyclo AB, Stockholm, Sweden; Purdue University, UNITED STATES

## Abstract

**Aim:**

To investigate the probability of marriage and divorce among Swedish military veterans deployed to Afghanistan relative to non-deployed matched comparators.

**Study design and setting:**

Matched cohort study in Sweden.

**Participants:**

Military veterans were identified through Swedish military personnel registers regarding foreign deployments, and comparators from the Military Service Conscription Register (1969–2013). Of 1,882,411 eligible conscripts, 7041 had served in Afghanistan at some point in time between 2002 and 2013. To each military veteran, up to 5 non-deployed comparators who underwent conscription were matched by age, sex, psychological assessment, cognitive ability, psychiatric history and social characteristics. After matching there were 4896 (82%) unmarried and 1069 (18%) married deployed military veterans. The main outcome was marriage or divorce after deployment to Afghanistan. Data on marital status were retrieved from Statistics Sweden until December 31, 2014.

**Results:**

During a median follow-up of 4.1 years after deployment of married individuals, 124 divorces were observed among deployed military veterans and 399 in the matched non-deployed comparator cohort (277 vs. 178 per 10,000 person-years; adjusted hazard ratio 1.61, 95%CI 1.31–1.97). During a median follow-up of 4.7 years after deployment in the unmarried cohort, 827 new marriages were observed among deployed military veterans and 4363 in the matched non-deployed comparators cohort (399 vs. 444 per 10,000 person-years; adjusted hazard ratio 0.89, 95%CI 0.83–0.96).

**Conclusion:**

Military veterans were more likely to divorce and less likely to marry after deployment compared with matched non-deployed comparators.

## Introduction

There is a general belief that military veterans struggle with intimate relations and re-adjustment to family life after returning from foreign deployment.[1,2] While there is agreement about the negative impact of combat exposure and post-traumatic stress disorder (PTSD) on romantic relations and family life,[1,3–6] the effect of foreign military deployment on marriage is debated.[1,2,7,8] A systematic review and a study of the entire male active US military population between the years 1998 and 2005 found that divorce rates among US military service members appear to be unchanged by the military operations in Afghanistan (Operation Enduring Freedom) and Iraq (Operation Iraqi Freedom), when comparing divorce rates before versus after the wars started, as well as in direct comparison with civilians matched by age, ethnicity, employment status, and education.[1,9] In the study by Negrusa et al of US military veterans, an increased divorce risk by deployment duration was found after several confounding factors were accounted for, an effect which was more pronounced for women.[8] Among veterans with PTSD symptoms after deployment, the divorce risk was increased further.[5]

European veterans from the recent wars in Iraq and Afghanistan generally have lower levels of PTSD than their US counterparts, which could be at least partly explained by differences in combat exposure during deployment.[10–14] It is therefore reasonable to assume limited post-deployment consequences on marriage and divorce among European veterans, given the unchanged divorce rates among US veterans. However, no such investigation among European veterans has yet been published in the scientific literature.

According to a systematic review by the RAND Corporation, there is a gap of research using longitudinal data investigating military deployment and risk of divorce, and studies with appropriate control groups.[1] This is not unique for investigations of divorce risk among deployed military veterans but a problem which has been identified by systematic reviews also for other outcomes.[15] Furthermore, the prospects for single military veterans of getting married after deployment have rarely been investigated, although these marriage prospects are important for young men and women who enter into military service (S1 Table). In the US, for example, married military veterans gain certain material benefits. [9]

The purpose of this study was to investigate the effect of deployment to Afghanistan on the post-deployment incidence of marriage and divorce among Swedish military veterans.

## Method

This is a cohort study of Swedish military personnel deployed to Afghanistan (deployed military veterans) and matched comparators without deployment history. Matched comparators were identified from the Military Conscription Service Register containing individuals who had gone through military conscription tests but not necessarily completed military service (S2 Table). The cohorts were identified and outcome data collected by linking nationwide Swedish registers by use of the unique personal identity number assigned to each Swedish resident. The study was approved by the Regional Ethics Committee in Stockholm (in Swedish: Regionala etikprövningsnämnden i Stockholm; board of medicine https://www.epn.se/stockholm/om-naemnden/), Sweden. Data were de-identified prior to delivery to the research group.

### Setting

In Sweden, military conscription was mandatory for men until 2010. Women volunteers and men went through a 2-day test protocol at military conscription, including both physical and psychological examinations. Only 2–3% of all Swedish men were exempt from conscription testing until 2005, in most cases because of severe disabilities or congenital disorders. Approximately two thirds of those who underwent conscription were accepted for military service with 7–15 months of military training depending on position. After their training, the military servicemen and women went back to civilian life. After 2005, the number of conscripts who were tested dropped to about a third of each birth cohort, and approximately one third of tested men and women were accepted for military service. Due to political decisions, mandatory conscription and military service was abolished in 2010 when Sweden began a transition to professional armed forces.

Men and women who completed their military service with sufficiently high military grades could apply for foreign military deployment at any time after their military service. The grades were set by the closest military officer in command and were approved by a ranking officer at the battalion level. The final decision as to who should be deployed was made by a military officer at the squadron level in the deployed military unit.

In December 2001, the NATO-led security mission International Security Assistance Force (ISAF) was established by the United Nations Security Council to improve the security situation in Afghanistan. The Swedish Armed Forces sent troops to ISAF from 2002 until 2014, when ISAF was disbanded. With over 8000 deployments, ISAF was the most extensive military engagement for Swedish forces during the period.

### Data sources

**Fig 1:**
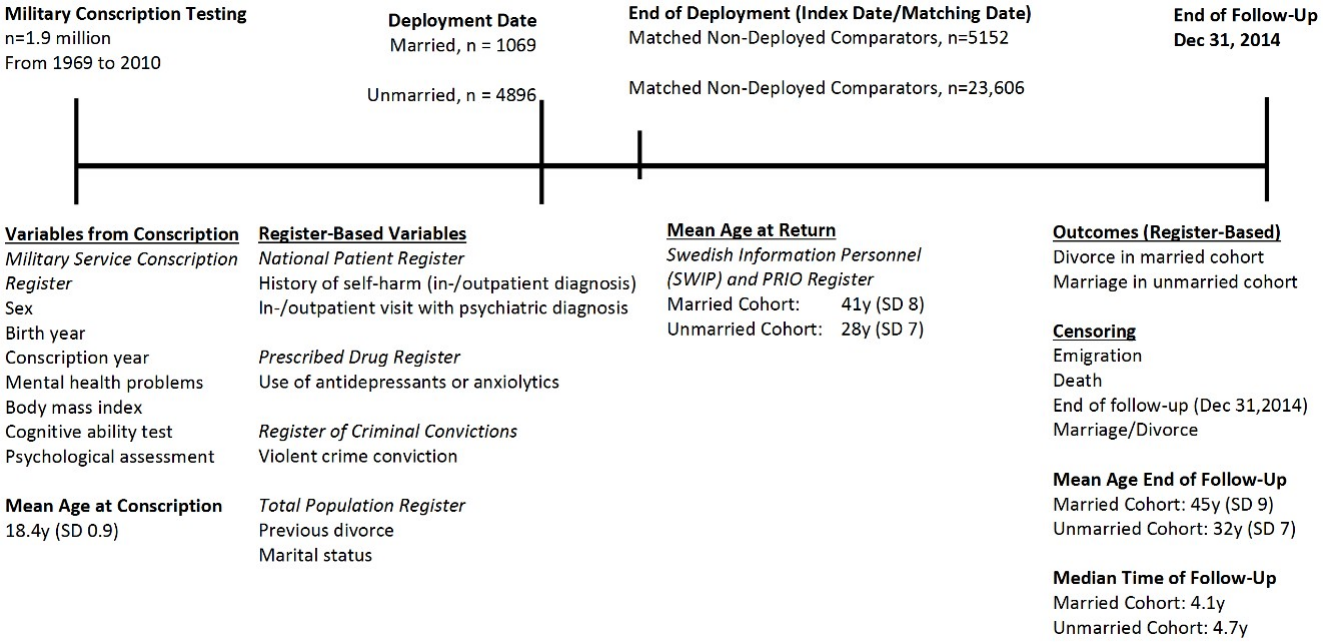
Timeline describing the cohorts, data sources and variables.

Data were collected from several government agencies. We used the following registers (Fig 1):

The Military Service Conscription Register: The coverage and use of the Military Service Conscription Register has been described elsewhere.[16] We retrieved variables on cognitive ability, psychological assessment, mental health, and BMI from the register. For assessment of cognitive ability, verbal, spatial, logic inductive, and technical ability were tested. The four different scores were converted to a Stanine scale (a scale from 1 to 9 with a normal Gaussian distribution) by the conscription authorities and combined into a single G-factor also on a Stanine scale. The psychological assessment was based on a questionnaire followed by an individual interview by a psychologist using a semi-structured interview form, to assess the ability of the conscripts to cope with prolonged and elevated stress, their leadership skills, and their suitability for military service.[17] Results were also reported on a Stanine scale. History of mental health problems was retrieved from a self-reported questionnaire (yes/no). Conscripts also had their height and weight measured which was used to calculate BMI (kg/m^2^; categorized into underweight (BMI<18.5), normal weight (BMI 18.5 to <25), overweight (BMI 25 to <30), and obesity (BMI≥30)).

Swedish Military Information Personnel (SWIP) Register: Deployment data were retrieved from the SWIP register for individuals deployed to Afghanistan at some point between 2002 and 2013. The SWIP register includes all types of service members, armed and unarmed, and contains information about country of deployment, date of deployment and return, sex, age, military rank, and regiment. Deployed military personnel have been registered in SWIP since 1965 except individuals serving in the Special Forces and classified personnel (representing <1%; personal communication, Swedish Armed Forces research coordinator Anders Claréus, June 5, 2015). Since 2012, the same deployment information has instead been entered into the *PRIO database*.

Total Population Register: Date of emigration was retrieved from this register along with marital status, date of marriage, date of divorce, and date of death.[18]

National Patient Register: This register was started in 1964 and attained nationwide coverage for inpatient care in 1987.[19] In 2001, hospital-based outpatient care was added. Through the National Patient Register, we retrieved data regarding mental health problems before deployment defined as any recorded psychiatric diagnosis (ICD9: 290–315; ICD10: F00-F99) after 1987 and any visit listing a self-harm diagnosis (ICD9: E950-E959, E980-E989; ICD10: X60-X84, Y10-Y34) after 1987.

Prescribed Drug Register: In July 2005, Sweden launched a nationwide complete prescribed drug register containing information regarding date and type of filled prescriptions.[20] From this source, we retrieved data on whether participants had one or more filled prescriptions for antidepressants (N06A) or anxiolytics (N05B) at any time prior to deployment.

The Register of Criminal Convictions: This register was started in 1973 and includes information based on convictions retrieved from all lower courts in Sweden. The register provides information on when a person was convicted of a felony and the type of sentence. We retrieved data on all types of violent crime convictions before deployment.

### Study population

Two cohorts were identified for this study from men and women in the Military Service Conscription Register.

Deployed Military Veterans: Swedish military personnel who deployed to Afghanistan at some time between January 1, 2002, and December 31, 2013 were identified through the SWIP register and the PRIO database.

Matched Non-Deployed Comparators: Up to 5 non-deployed comparators were selected by an exact matching procedure from individuals who underwent conscription. Matching factors included sex, deployment factors (age [±1 year], marital status [married/unmarried]), conscription data (conscription year, cognitive ability [1–9], psychological assessment [1–9], self-reported mental health problems [yes/no], and BMI [<18.5/18.5 to <25/25 to <30/≥30]), pre-deployment history of violent crime convictions (yes/no), divorce (yes/no), deliberate self-harm (yes/no), and one or more prescription fillings of antidepressants or anxiolytics (yes/no). The matching date was set to each veteran´s homecoming date. Individuals who had deployed before 2002 or elsewhere than Afghanistan were not eligible as comparators.

### Outcome and follow-up

Date of legal marriages and divorces were retrieved from the Total Population Register until December 31, 2014. Start of follow-up was the day of return to Sweden after deployment, for each deployed military veteran and their non-deployed comparators. Participants were followed until date of event (marriage for unmarried participants; divorce for married participants), emigration, death, or end of follow-up, whichever came first (Fig 1).

### Statistics

Pre-deployment and conscription characteristics were compared between deployed military veterans and non-deployed comparators using ANOVA for continuous variables and logistic regression for categorical variables to account for the matched design. This comparison and all other analyses were performed conditioned on the matching set consisting of one deployed military veteran and up to 5 non-deployed comparators sharing the same individual matching characteristics.

Incidence rates and Kaplan-Meier failure functions were used to present absolute probabilities. Cox proportional hazards models were used to estimate hazard ratios for (a) the probability of divorce among married participants and (b) the probability of marriage among unmarried participants.[21] Additional adjustment was made for history of healthcare visits listing a psychiatric diagnosis prior to deployment (or corresponding matching date for comparators) using data from the nationwide National Patient Register. When analysing the probability of divorce, adjustment for duration of marriage was made.

Sensitivity analyses were conducted excluding a) women, b) deployed military veterans with deployment duration <60 days, c) individuals who married the year before deployment (the divorce outcome) or individuals with a history of divorce (the marriage outcome), d) individuals who went through conscription after 2006 (when the Swedish conscription system changed) and e) military veterans deployed in unarmed service such as priests or chefs.

Data were analyzed using SAS (version 9.4, SAS Institute, Cary, NC) and Stata (version 13.0, College Station, TX). All tests were two-sided and P-values <0.05 were considered statistically significant.

## Results

### Participant characteristics

We identified 6072 military veterans with deployment to Afghanistan between January 1, 2002, and December 31, 2013. Of those, 107 were not eligible for matching due to missing data in the Military Service Conscription Register (S3 Table). After matching, 1069 married (18%) and 4896 (82%) unmarried deployed military veterans remained.

Compared to age- and sex-matched non-deployed comparators, deployed military veterans had higher scores on cognitive ability and psychological assessment (S1 Fig), lower prevalence of antidepressant and anxiolytic medication use, as well as lower prevalence of history of self-harm and violent crime convictions (S4 Table). This selection of healthier individuals for military deployment was eliminated by the matching procedure which used all these individual traits as matching factors.

Married vs. Unmarried Deployed Military Veterans Married military veterans had a higher average score on the cognitive ability test (72% vs. 60% scoring 6 or more) and the psychological assessment at conscription compared to their unmarried counterparts (83% vs. 78% scoring 6 or more; S1 Fig).

Married military veterans were also older at deployment than unmarried military veterans (mean age 41 vs. 26 years; Table 1). Possibly due to age, married military veterans had higher pre-deployment proportions of registered self-harm (1.7% vs. 1.3%), use of antidepressants or anxiolytics (4.6% vs. 3.0%), and were more likely to have divorced previously in comparison to unmarried military veterans (7.6% vs. 2.9%). There was no difference in deployment duration between married and unmarried deployed military veterans (mean 5.8 months; Table 1).

**Table 1:** Description of deployed military veterans and the matched non-deployed comparators at conscription and before deployment to Afghanistan.

	Married at Deployment		Unmarried at Deployment	
	Deployed Military Veterans	Matched Non-Deployed Comparators	Deployed Military Veterans	Matched Non-Deployed Comparators
**N**	1069	5152	4896	23,606
Men	1054 (99%)	5110 (99%)	4639 (95%)	22,569 (96%)
Women	15 (1.4%)	42 (0.82%)	257 (5.3%)	1037 (4.4%)
**At Conscription; Mean (SD)**				
Age (Years)	18 (1)	18 (1)	18 (1)	18 (1)
Height (cm)	180 (7)	180 (7)	180 (8)	180 (7)
Weight (kg)	72 (10)	72 (10)	73 (11)	73 (11)
Body Mass Index (kg/m^2^)	22.3 (2.3)	22.3 (2.7)	22.6 (2.8)	22.7 (2.8)
History of Mental Health Problems	9 (0.84%)	38 (0.74%)	12 (0.25%)	55 (0.23%)
**Deployment**				
Age at Deployment (Years), Mean (SD)	40 (8)	40 (8)	27 (7)	27 (7)
Divorce	81 (7.6%)	387 (7.5%)	141 (2.9%)	653 (2.8%)
Healthcare Contact with Psychiatric Diagnosis	79 (7.4%)	350 (6.8%)	326 (6.7%)	1490 (6.3%)
History of Antidepressant or Anxiolytics Use	49 (4.6%)	220 (4.3%)	146 (3.0%)	625 (2.7%)
History of Self-Harm	18 (1.7%)	59 (1.2%)	61 (1.3%)	229 (1.0%)
Violent Crime Conviction	26 (2.4%)	118 (2.3%)	78 (1.6%)	329 (1.4%)
Duration of Deployment (Months), Mean (SD)	5.8 (1.8)	N/A	5.8 (1.8)	N/A
Duration of Marriage (Years), Mean (SD)	8.9 (8.1)	10.0 (7.9)	N/A	N/A

Data are mean (SD) for continuous variables and n (%) for categorical variables. All individuals in the matched cohort were required to have gone through military conscription testing but were not required to have completed military service. Psychiatric diagnosis: ICD9 290–315 and ICD10 F00-F99; Deliberate self-harm: ICD9 E950-E959, E980-E989 and ICD10 X60-X84, Y10-Y34. Antidepressants: ATC-code NO6A; Anxiolytics: ATC-code N05B. Pre-deployment refers to history allowed by the register in use. Matching factors included sex, deployment factors (age, marital status), conscription data (conscription year, cognitive ability, psychological assessment, self-reported mental health problems [yes/no], and BMI), and pre-deployment history of violent crime convictions, divorce, deliberate self-harm, and one or more prescription fillings of antidepressants or anxiolytics.

Deployed Military Veterans vs. Matched Non-Deployed Comparators Due to the matching procedure, there were no statistically significant differences between the deployed military veterans and the matched non-deployed comparators in any of the matching variables (Table 1). History of healthcare visits listing a psychiatric diagnosis was not a matching factor, but there was no statistically significant difference in this variable between deployed military veterans and matched non-deployed comparators before the matching date. Deployed military veterans had statistically significantly shorter duration of marriage (8.1y vs. 10.0y; P <.0001).

### Probability of divorce

**Fig 2:**
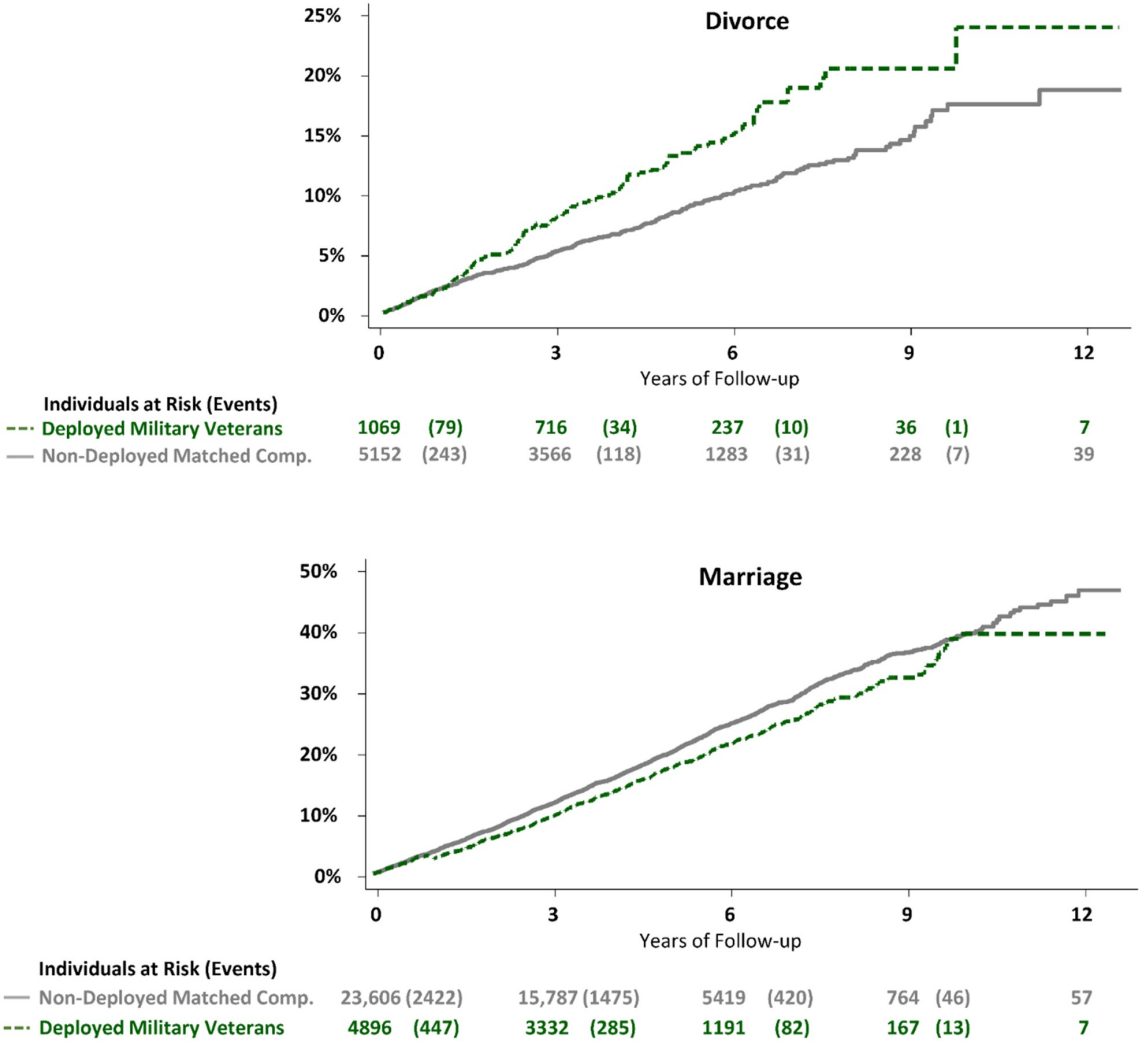
(upper panel) Cumulative incidence of divorce among deployed military veterans and non-deployed matched comparators who were married at time of deployment (or matching). (lower panel) Cumulative incidence of marriage among deployed military veterans and non-deployed matched comparators who were unmarried at time of deployment (or matching).

**Fig 3:**
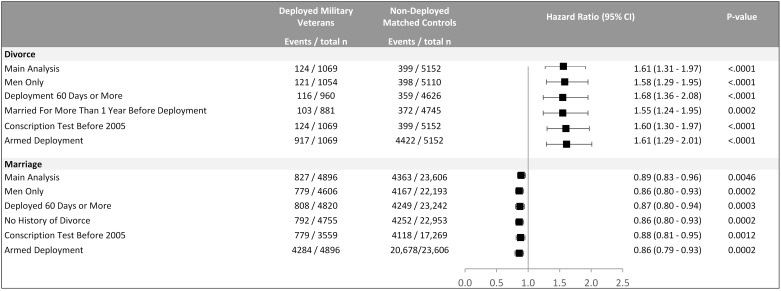
Adjusted hazard ratios for marriage and divorce among deployed military veterans and matched comparators.

During a median follow-up of 4.1 years after returning from Afghanistan, the incidence of divorce per 10,000 person-years was 277 for deployed military veterans and 178 for matched non-deployed comparators (adjusted hazard ratio 1.56, 1.27–1.91; P <.0001; Figs 2 and 3). Duration of marriage was associated with divorce but did not alter the main results when adjusted for in the model (adjusted hazard ratio 1.61, 1.31–1.97; P <.0001).

### Probability of marriage

During a median follow-up of 4.7 years after returning from Afghanistan, the incidence of marriage per 10,000 person-years was 399 for deployed military veterans and 444 for matched non-deployed comparators (0.89, 0.83–0.96; P = 0.0046; Figs 2 and 3).

### Sensitivity analyses

The adjusted hazard ratios for divorce or marriage were largely unaffected when restricting the analyses to men only, deployments ≥60 days, individuals married for more than 1 year prior to deployment (for the divorce outcome) or having no history of divorce (for the marriage outcome), individuals having performed conscription testing before 2006, and military veterans deployed in armed service (Fig 3).

## Discussion

This study investigated the association of deployment to Afghanistan within ISAF and the post-deployment incidence of marriage and divorce among Swedish military veterans. We found that the military veterans who were unmarried at deployment (82%) were less likely to marry, and that the military veterans who were married (18%) were more likely to divorce after deployment in comparison to matched non-deployed comparators.

### Previous research

Previous US studies have found mixed results for the effects of military deployment on divorce. US veterans serving in Vietnam, for example, do not seem to have an increased divorce rate than Americans in general, even though a strong association between PTSD and divorce has been reported in this population.[2,4] Studies on US Gulf War veterans have reported no reduction in marital satisfaction following deployment. A recent study based on data from the entire US military population from 1998 to 2005 could not detect any difference in divorce or marriage rates between veterans deployed to Iraq or Afghanistan and a comparison group from the general population matched on age, ethnicity, employment status, and education.[9] Other studies have found that additional deployment months do increase the divorce hazard, that this effect is more pronounced among women, and that the risk of divorce is further elevated among veterans who report symptoms of PTSD after deployment.(5,8).

A limitation of these US studies is that they did not take into account pre-deployment mental health, a factor that is likely to be linked both to the exposure (military deployment) and the outcome (marriage/divorce). In our study, individuals who went through the selection process for foreign military deployment had considerably better mental health (S1 Fig) than the general population (data not shown). Irrespective of potential differences in mental health, it is likely that direct comparisons between countries are not straightforward considering the differences between the US studies and the present study in terms of populations and context, especially the work-related benefits associated with marriage for active US service members such as economic supplements for housing and living expenses as well as healthcare coverage for spouses.[9] Such conditional benefits are not available in Sweden. To the best of our knowledge, no comparable European studies have yet been published.

### Mechanisms

Meadows et al have suggested that higher divorce rates following foreign military deployment may be caused by traumas and unresolved mental health problems.[22] For the current cohort of Swedish military veterans deployed to Afghanistan, this explanation is less likely due to the low suicide and nonfatal self-harm rate found in a previous study.[23] Although severe mental health problems may not explain the increased risk of divorce that we found, a meta-analysis has reported that alcohol and substance abuse is more prevalent in military veterans from the Iraq, Afghanistan and Gulf wars than in the civilian population, and such abuse may influence the risk of divorce. Unfortunately, it is difficult to obtain reliable data on alcohol and substance abuse, and alcohol abuse is likely related to mental illness.

Another possible explanation could be that several months of work-related absence from home hampers the possibility to nurture an ongoing marriage. Furthermore, some individuals could also decide to serve with the armed forces in order to distance themselves from an already failing marriage.

We ruled out a number of potential mechanisms behind our findings by restricting the cohort of deployed military veterans under investigation. We did not see any material changes in the magnitude of the hazard ratios when excluding women, veterans with short deployment duration (<60 days), recently married individuals (the year before deployment), individuals with history of divorce, and unarmed service members. Also, duration of marriage was associated with divorce rate and there were statistically significant differences between deployed military veterans and matched comparators in our study. Adjusting for duration of marriage had, however, only a limited effect on the hazard ratio estimate.

Finally, the differences in divorce and marriage rates found between the military veterans and the non-deployed matched comparators from the general population could be driven by personality differences. The individuals who volunteer to military service in Afghanistan, with all the associated risks and hardships, might have a more adventurous character and be less risk averse than the comparators who chose not to volunteer for foreign military service. Individuals who are more prone to risk taking may be less interested in as well as less suited for marriage. Hooper et al have proposed that voluntarily entering the military brings together individuals with high risk tolerance, which is desirable for certain military occupations.

### Strengths

One major strength of this study was the possibility to compare deployed military veterans with appropriate individuals from the general population. A systematic review performed by the RAND Corporation (a nonprofit research organization) highlighted the need for new studies with suitable control groups in the research field.[1] The present study used a control group matched on age, sex, history of divorce, cognitive ability, psychological assessment, mental health problems prior to deployment (including but not restricted to self-harm and use of antidepressants or anxiolytics), and a history of violent crime convictions. This extensive matching procedure is likely to have reduced the impact of the selection bias referred to as the “healthy soldier effect”, representing the physical and psychological selection preceding deployment which results in soldiers being both physically and mentally healthier than people in the general population who have not gone through similar testing protocols. Another strength was the outcome ascertainment, where the present study had several years of complete follow-up through nationwide registers of marriages and divorces.

### Limitations

A limitation of this study was the lack of detailed information about divorce such as marriage quality prior to deployment, the reason for divorce, and the initiator of the divorce. Another limitation is that we only investigated marriages and divorces, but did not have data on cohabiting men and women who were not married or did not marry. In the deployment data, there was no information regarding combat exposure, neither at the individual nor at the aggregate level, precluding analyses stratified by a factor that has previously been shown to influence marriages.[5] Regarding healthcare visits with psychiatric diagnoses, data were available from the National Patient Register on hospital-based inpatient and outpatient care, but no data from primary care were available. However, primary care visits resulting in prescriptions were captured via the nationwide Prescribed Drug Register whenever a prescription was filled.

### Implications

The deployed military veterans and their families should be informed prior to deployment that divorce is more common after foreign military deployment than in comparable non-deployed individuals in the general population, at least in a Nordic context. Future research should try to shed light on the mechanisms behind the higher divorce rate among deployed military veterans, for example by collecting information on the quality of marriages before deployment, and, in the event of a divorce after deployment, information about which partner initiated the divorce and for what reason. Preemptively informing about where to find family therapists when soldiers return from deployment may also be warranted.

### Conclusions

Swedish military veterans who served in Afghanistan within ISAF were more likely to divorce and less likely to marry after deployment compared to non-deployed civilian comparators with similar demographic and pre-deployment mental health status. These findings stand in contrast to many studies with non-deployed comparators among US veterans who served in Vietnam, Afghanistan or Iraq.

## Supporting information

S1 Table Description of the deployed military veterans and the comparator cohort. (TIF)

S2 Table Evidence table regarding deployed military veterans and marital status. (TIF)

S3 Table Deployed military veterans with and without conscription data. (TIF)

S4 Table Description of three cohorts Deployed military veterans, fully matched cohort and age- and sex- matched comparators. (TIF)

S1 Fig Cognitive ability and psychological assessment score for unmarried and married deployed military veterans and age- and sex-matched comparators. (TIF)
