# Supplementary figures and images for: Marriage and divorce after military deployment to Afghanistan: A matched cohort study from Sweden
Source: PLoS One. 2019 Feb 1;14(2):e0207981. doi: 10.1371/journal.pone.0207981 (PMC6358058; doi:10.1371/journal.pone.0207981)

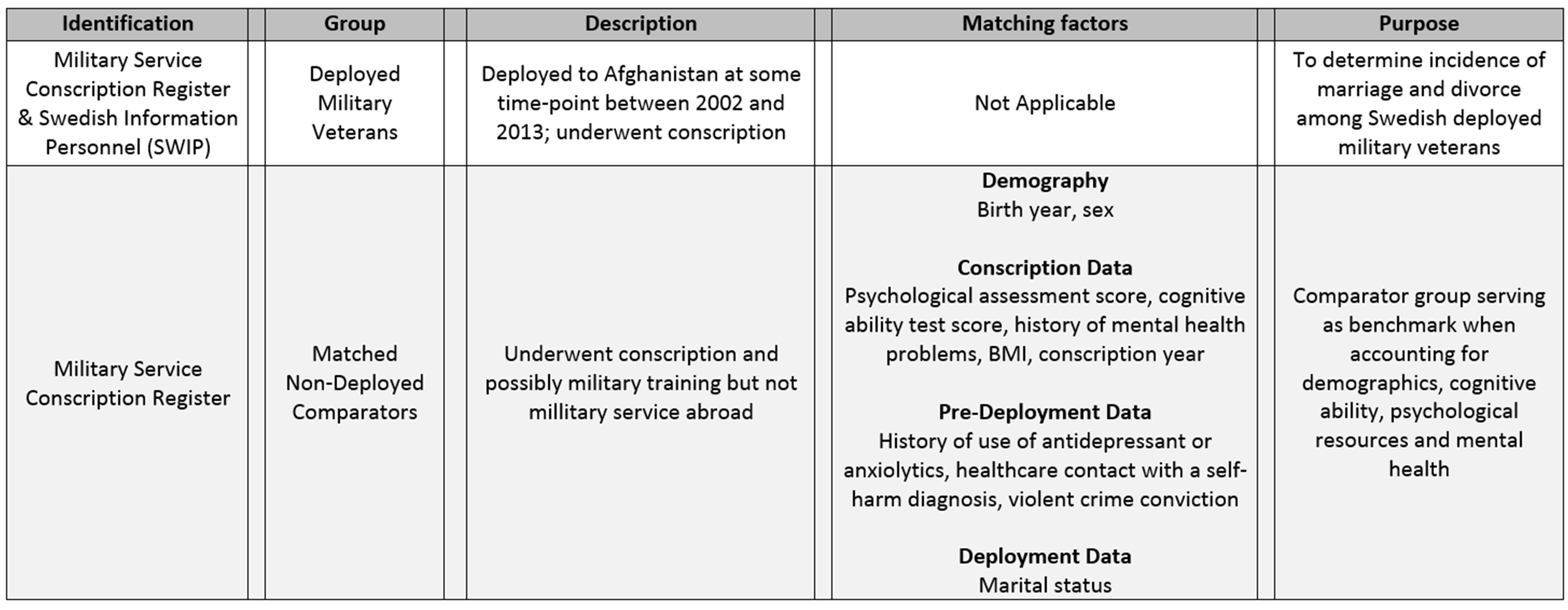

Supplement: S1 Table — (TIF) [file pone.0207981.s001.tif]

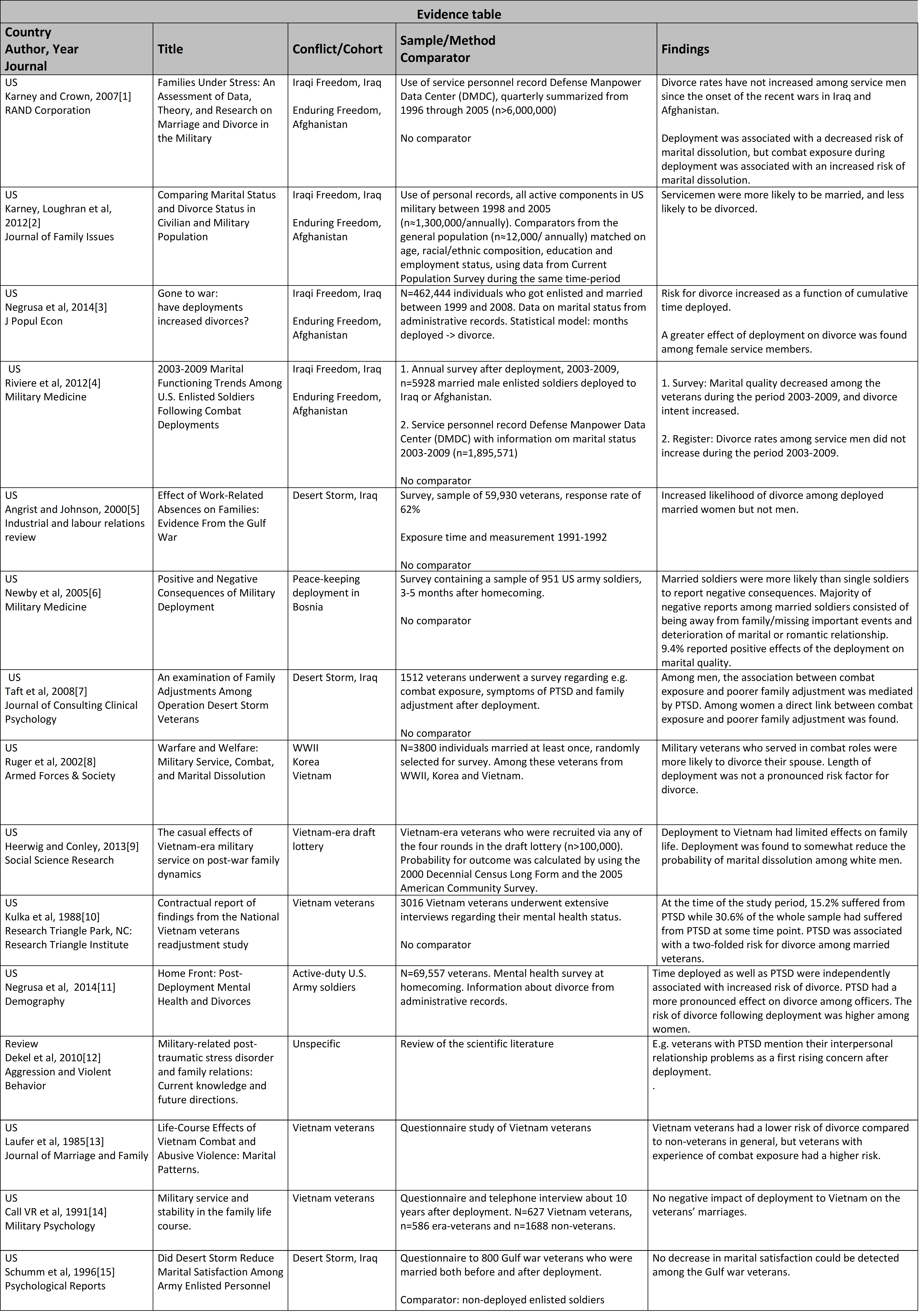

Supplement: S2 Table — (TIF) [file pone.0207981.s002.tif]

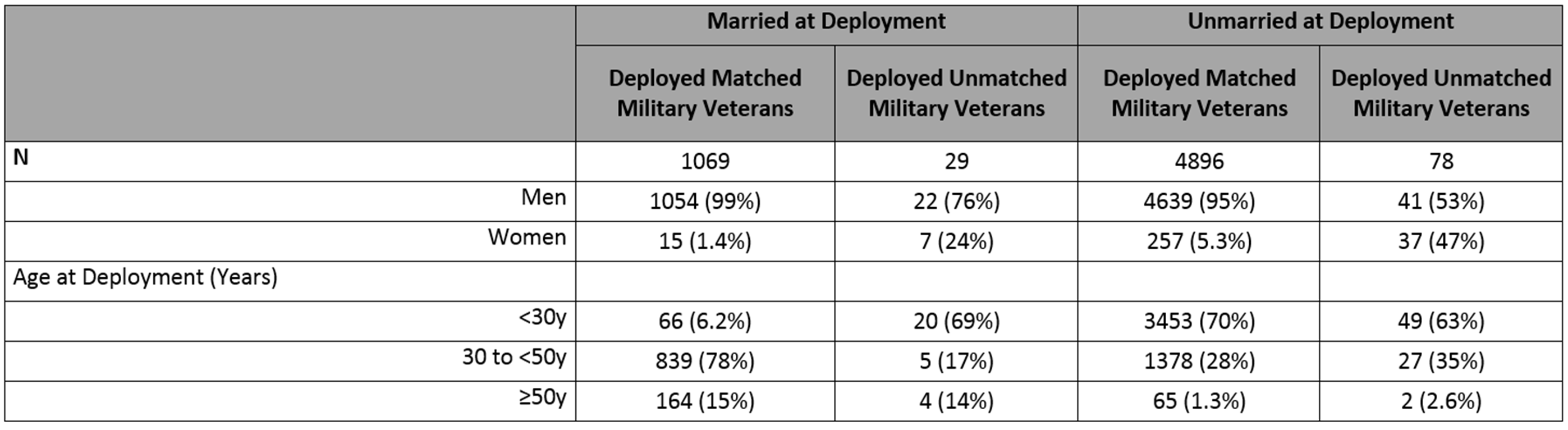

Supplement: S3 Table — (TIF) [file pone.0207981.s003.tif]

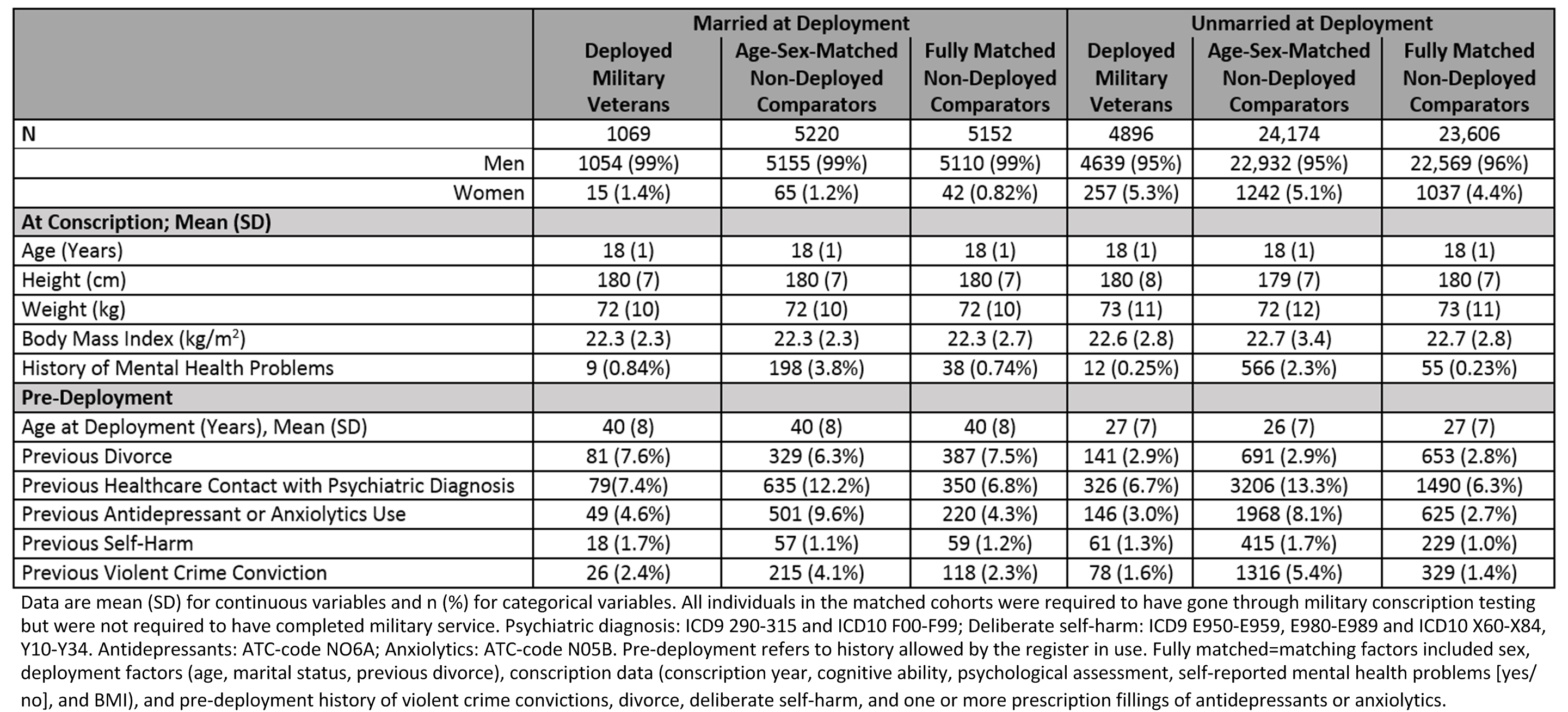

Supplement: S4 Table — (TIF) [file pone.0207981.s004.tif]

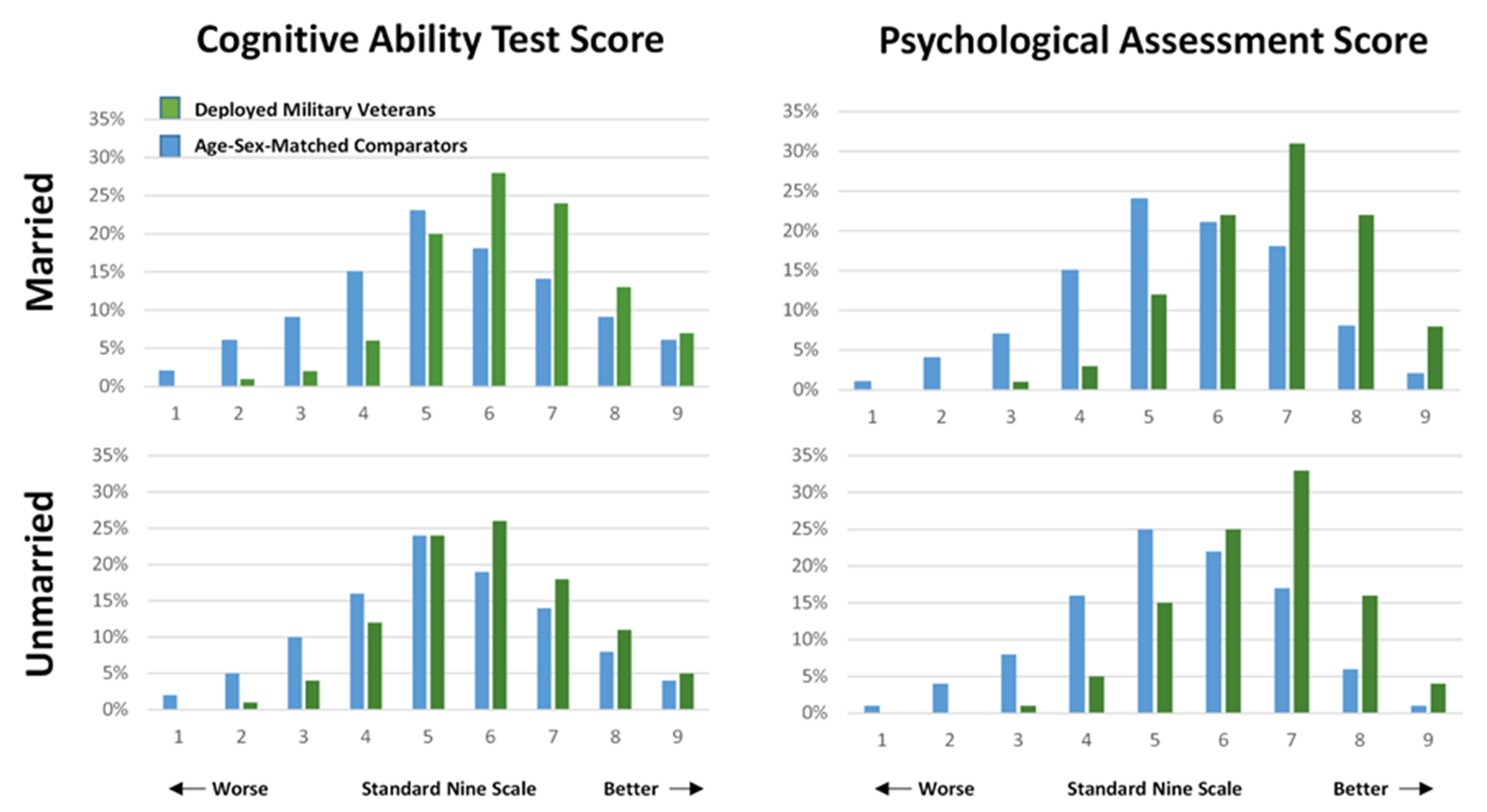

Supplement: S1 Fig — (TIF) [file pone.0207981.s005.tif]
